# Early Summer Drought Stress During the First Growing Year Stimulates Extra Shoot Growth in Oak Seedlings (*Quercus petraea*)

**DOI:** 10.3389/fpls.2016.00193

**Published:** 2016-02-23

**Authors:** Arion Turcsán, Kathy Steppe, Edit Sárközi, Éva Erdélyi, Marc Missoorten, Ghislain Mees, Kristine V. Mijnsbrugge

**Affiliations:** ^1^Research Institute for Nature and ForestGeraardsbergen, Belgium; ^2^Department of Biometrics and Agricultural Informatics, Corvinus University of BudapestBudapest, Hungary; ^3^Department of Forest Reproductive Material and Plantation Management, Institute of Silviculture and Forest Protection, University of West HungarySopron, Hungary; ^4^Laboratory of Plant Ecology, Department of Applied Ecology and Environmental Biology, Ghent UniversityGhent, Belgium; ^5^Department of Soil Science and Water Management, Corvinus University of BudapestBudapest, Hungary; ^6^College of Commerce, Catering and Tourism, Budapest Business SchoolBudapest, Hungary; ^7^Agency for Nature and ForestBrussels, Belgium

**Keywords:** drought, oak seedling, apical bud, shoot growth, re-watering, general linear mixed models

## Abstract

More severe summer droughts are predicted for mid-latitudes in Europe. To evaluate the impact on forest ecosystems and more specifically on forest regeneration, we studied the response to summer drought in oak seedlings (*Quercus petraea*). Acorns were collected from different mother trees in three stands in Belgium, sown in pots and grown in non-heated greenhouse conditions. We imposed drought on the seedlings in early summer by first watering the pots to saturation and then stopping any watering. Weight of the pots and stomatal conductance were regularly measured. Re-watering followed this drought period of 5 weeks. Height of the seedlings and apical bud development were observed. Stomatal resistance increased toward the end of the experiment in the drought-treated group and was restored after re-watering. The seedlings from the drought treatment displayed a higher probability to produce additional shoot growth after re-watering (*p* ≤ 0.05). A higher competition for water (two plants per pot) increased this chance. Although this chance was also higher for smaller seedlings, the actual length of the extra growth after re-watering was higher for larger seedlings (*p* ≤ 0.01). Both in the drought-treated and in the control group the autochthonous provenance growing on a xeric site produced less extra shoots compared to the two other provenances. Finally, stressed plants showed less developed apical buds compared to the control group after re-watering, suggesting a phenological effect on the growth cycle of oaks (*p* ≤ 0.0001). The higher chance for an extra shoot growth after the drought period can be considered as a compensation for the induced growth arrest during the drought period.

## Introduction

Several periods of dry, wet, cold, or warm climate were recorded during the past centuries in Europe (Masson-Delmotte et al., 2005). Furthermore, the number of precipitation anomalies increased during the past century (Zhang et al., 2007). The predicted climate change indicates even more extreme weather events such as longer dry periods, swifts in precipitation and rain intensity. Forest vitality will be challenged by such changes and forests will become more vulnerable not only in Europe (Lindner et al., 2010), but all over the globe (Choat et al., 2012). For Europe, climate projections predict increasing temperatures and irregular precipitation patterns in summer, augmenting the number and the intensity of drought events (IPCC, 2013). For Belgium, drier conditions due to climate change are predicted for the end of the century especially in summer periods (Baguis et al., 2010).

A large part of the forests in the lower countries grows on sandy soils which are characterized by a relatively low water holding capacity, making them especially vulnerable to extreme drought events during the growing season (Van der Werf et al., 2007). Soil water shortage results in leaf stomatal closure, which limits leaf cooling and is quickly followed by leaf damage (Bréda et al., 2006). Subsequently, drought stress disrupts water transfer in the xylem tissue through cavitation of vessels resulting in dying off of twigs and roots (Barigah et al., 2013). Stomatal closure also hampers carbon assimilation in the tree (McDowell, 2011). These physiological effects of drought stress restrict in the first place biomass production, and additionally increase susceptibility and vulnerability toward secondary stresses such as frost or fungal and insect attacks (Bréda et al., 2006; Lindner et al., 2010).

Sessile oak (*Quercus petraea*), pedunculate oak (*Quercus robur*) and beech (*Fagus sylvatica*) take great stake in European forest ecosystems (Mátyás, 1996). Oak species have a lower competition capacity compared to beech under natural conditions, but they adapt better to poor weather or soil conditions (Thomas and Gausling, 2000) and are well known for being relatively drought tolerant, because of their deep roots system and effective water transport (Landolt et al., 2010; Kuster et al., 2011). During the past centuries dry conditions in early summer restricted the growth of beech and oak trees (Pilcher and Gray, 1982; Dittmar et al., 2003; Lebourgeois et al., 2005), although oak species show a comparably lower sensitivity to drought at the more humid sites in Europe (Scharnweber et al., 2011). Stem diameter growth of adult beech trees is stronger reduced by dry summer periods compared to adult oak trees (Leuschner et al., 2001). In the Netherlands, oak and beech display similar growth patterns, suggesting similar influential environmental factors (Van der Werf et al., 2007). A relative strong correlation between intra-annual growth pattern and precipitation in this region indicates the importance of the latter for proper growth in both species (Van der Werf et al., 2007). Arend et al. (2011) pointed to the provenance-specific growth responses to drought of *Quercus* sp. with shoot height growth being more negatively affected by drought in northern provenances compared to more southern provenances within Switzerland.

*Quercus* species are characterized by a cyclic growth. The buds flush several times within the growing season, each time followed by distinctive rest periods, with a trade-off between root and shoot growth (Reich et al., 1980; Harmer, 1990). The auxin/cytokinin ratio plays an important role in the induction of bud dormancy and bud burst (Cline and Harrington, 2006; Su et al., 2011; Vanstraelen and Benková, 2012). Under uniform growing conditions buds flush, and thus shoots and leaves grow, and subsequent rest periods occur very synchronous among oak seedlings (Reich et al., 1980). Also, oak seedlings commonly display multiple flushing in spring when environmental conditions are not limiting (Reich et al., 1980).

Broadmeadow and Jackson (2000) observed an overall reduction in plant biomass induced by drought stress, but at the same time the root-shoot relation shifted with the oak (*Q. petraea*) saplings producing more roots compared to shoots under drought conditions. Furthermore, increase of fine root biomass in oak was found under water deficit, attempting to reach water supplies at lower soil layers (Thomas and Gausling, 2000). However, biomass (leaves and shoots) decreased in the long term under repeated drought stress (Broadmeadow and Jackson, 2000; Thomas, 2000) with diminished shoot growth being less severe in sessile oak (*Q. petraea*) compared to pedunculate oak (*Q. robur*; Fonti et al., 2013). Saplings from an oak provenance growing at a drier site showed higher biomass loss at water limiting conditions compared to a provenance from a more humid site, questioning the suitability of xeric provenances in mitigating predicted climate change (Kuster et al., 2013). Spieß et al. (2012) observed “compensated growth” induced by re-watering after one or two drought periods within the growing season. In their study, Spieß et al. (2012) reduced soil water conditions by 20–25% compared to the control groups, and observed reduced shoot growth during the drought treatment on 2–3 years old oak saplings from one genotype and a fourth shoot on some of the saplings after re-watering.

In this study, we investigated the immediate effects of a short drought stress in early summer on potted, 1-year old oak (*Q. petraea*) seedlings from three different provenances. As oak is characterized by a cyclic growth pattern, we focused on shoot growth and bud development. Competition for water between plants generally occurs when availability is reduced (Craine and Dybzinski, 2013). Intraspecific competition has impact on seedling performance and growing pattern (Shainsky and Radosevich, 1992). Seedlings were single or double in the pots in our experiment, adding an extra competition effect for the latter.

## Materials and Methods

### Source Material

Acorns were collected per mother tree at three different locations in Flanders (northern part of Belgium): Klaverberg (KLA, 51°0′57.8556′′N 5°31′57.0384′′E), Voeren (VOE, 50°45′31.5612′′N 5°45′39.9348′′E) and Borgloon (BOR, 50°48′22.0680′′N 5°20′34.872′′E) at the end of October 2013. The three provenances differ in soil type and/or in stand history. Klaverberg is a small relict of oak coppice wood growing on inland sand dunes within a former heath land. The oaks here are most probably local of origin. The stand is characterized by a large structure diversity. Acorns were collected from the visually older coppice stools. As the oaks are mostly growing widely spaced, the chance on mixture of acorns from different mother trees was negligible. Voeren is a classical planted forest stand, even aged and approximately 80 years old, growing on a loamy soil type. The origin of the planted material is unknown. Borgloon is also a planted forest and approximately 100 years old. The forest stand grows on sandy soil and the origin of the planted material is also unknown. Acorns were collected underneath 14 mother trees from Voeren, 13 mother trees from Klaverberg, and three mother trees from Borgloon, which all showed a well-developed crown (dominant trees). Collection was performed only close to the stem, minimizing the chance on mixing acorns between different mother trees. Before sowing, a water-swimming test was used to assess the vitality of the seeds and the unhealthy seeds were removed. The seeds were further visually controlled. The checks resulted in 664 acorns from Klaverberg, 744 acorns from Voeren, and 154 from Borgloon.

### Experimental Design

In November 2013, the collected seeds were sown in forestry trays (two seeds per cell) using standard nursery potting soil. During winter, the trays were watered manually keeping the soil moist. The experiment was located in a greenhouse with automatic temperature regulation, keeping the greenhouse frost-free in wintertime, but without additional heating. The germinated seedlings were transferred in April 2014 to 1-l pots (12 cm × 11 cm × 11 cm) using standard nursery potting soil (Organic matter concentration 20%, pH 5.0–6.5, Electrical Conductivity (EC): 450 μS/cm, dry matter 25%, Fertilization: 1.5 kg/m^3^ powdered compound fertilizer NPK 12 + 14 + 24). Non-germinated seeds were removed, whereas double plants in one tray cell were kept together. The seedlings were kept without any additional fertilization during the experiment.

We choose the usage of seedlings in pots in our experiment, rather than working in a field experiment outdoor, as this allows to impose a drought period on a subset of plants while both treated and control plants can be subjected to very similar other growth conditions (light, temperature, nutrient availability). Furthermore, it allows monitoring indirectly the reduction in water availability by weighing of the pots. The set-up consisted of two main groups: the control group with 148 pots (66 single seedling and 164 double seedling per pot) with seedlings from KLA, 116 pots (52 single seedling and 128 double seedling per pot) from VOE and 37 pots (12 single seedling and 50 double seedling per pot) with seedlings from BOR, and the drought-treated group with 137 pots (57 single seedling and 190 double seedling per pot) containing seedlings from KLA, 124 (66 single seedling and 116 double seedling per pot) from VOE and 43 (10 single seedling and 66 double seedling per pot) from BOR. All germinating plants were given water at regular times according to the visual needs of the pots as judged by experienced greenhouse workers. In both groups, the three provenances were individually mingled at random (completely randomized).

On May 15, 2014 the two groups of plants were soaked overnight in a water basin with a water level up to two cm at the bottom of the pots. In this way all the pots were fully saturated with water. Up to July 1, 2014 the drought-treated group was not watered anymore, whereas the control group was further watered according to the visual needs of the plants. All plants were re-watered on July 2, 2014 by soaking the two groups of plants in the same water basin in the same way. After this, both groups were kept in well-watered conditions according to the visual needs of the plants.

### Measurements and Scoring

During the drought treatment all pots were weighed every week to measure the water loss following the first water saturation treatment. As a proxy for the level of drought stress, the weight loss of the individual pots at the end of the treatment period was calculated relative to the initial weight at fully saturated condition. In the statistical models, drought stress was expressed as the weight loss of the last weighing of the individual pots at the end of the treatment, just before re-watering, divided by the weight of the individual pots at full saturation at the beginning of the treatment.

Thirty pots with relative high plants were randomly chosen from the control group as well as 30 from the drought-treated group to monitor the treatment effect. Leaf stomatal aperture in terms of leaf resistance to water vapor was measured weekly with a diffusion porometer (Model AP4, Delta-T Devices, Burwell, Cambridge, UK) during the entire drought period. As stomata are sensitive to drought stress, high resistance values represent a closing reaction (Schulze et al., 1972), and declining stomatal conductance and leaf assimilation rate (Farquhar and Sharkey, 1982). The porometer measurements were conducted during daytime between 10 a.m. and 3 p.m.

The height of the seedlings was measured with a ruler at the end of the drought treatment and at full recovery of the plants (on September 4, 2014).

The apical bud of the highest plant per pot was scored on August 28, 2014, following a binary scoring system with buds well developed and colored brown, as opposed to any other stage of bud development (bursting, absent or small and green).

### Data Analysis

The open source software R 3.1.2 (R development Core Team, Vienna, Austria) was used for all statistical analyses. Three response variables were modeled using (generalized) linear mixed models.

As a larger part of the plants did not show any height growth between July 1, 2014 and September 4, 2014, a first binary response variable was deduced from the height data indicating no growth or growth between the two time points. From all plants that had grown between the two height measurements, the height increment was calculated as a second continuous variable. Finally the apical bud score was the third binary response variable. The first and third response variables were modeled using logistic regression (generalized linear mixed models) in the package lme4 (Bates et al., 2014), whereas the continuous response variable was examined using linear regression (linear mixed model) in the package nlme (Pinheiro et al., 2011). In all three models, the same covariates were checked for significant explanatory power: the height immediately after the drought treatment (continuous variable), the provenance of the seedlings (factor variable) and the number of seedlings per pot (factor variable). All three covariates were first included in each model with an interaction term with weight loss of the pots relative to the fully water saturated condition. Using drop 1, a likelihood ratio test (and a maximum likelihood estimation for the linear model of the continuous response variable height increment), the fixed part of the models was reduced up to only significant terms. In all models the mother plant from which acorns were collected was in the random part (random intercept). In addition, the linear model of height increment showed a better fit using a log transformation of the response variable. The predict command in lme4 and nmle was applied for drawing the regression curves. The number of seedlings used in the calculation are indicated in **Table 1**.

**Table 1:** Number of seedlings used in the models for extra shoot growth, height increment and bud development.

	Model I					Model II					Model III				
	Extra shoot (binary)					Height increment (continous)					Bud development (binary)				
	CON		STR			CON		STR			CON		STRy		
Sp	1	2	1	2	Sum	1	2	1	2	Sum	1	2	1	2	Sum
KLA	66	164	57	190	**477**	5	4	10	35	**54**	66	84	57	92	**300**
VOE	52	128	66	116	**362**	9	10	17	28	**64**	52	73	66	72	**263**
BOR	12	50	10	66	**138**	1	1	2	17	**21**	12	29	10	33	**84**
Total					**977**					**139**					**647**

*The type of response variable is indicated between brackets. CON: control group, STR: drought-treated group, Sp: number of seedlings per pot. The provenances are VOE, BOR, and KLA. The bolded values are the sum values of each line per model.*

## Results

### Stress Symptoms

**FIGURE 1:**
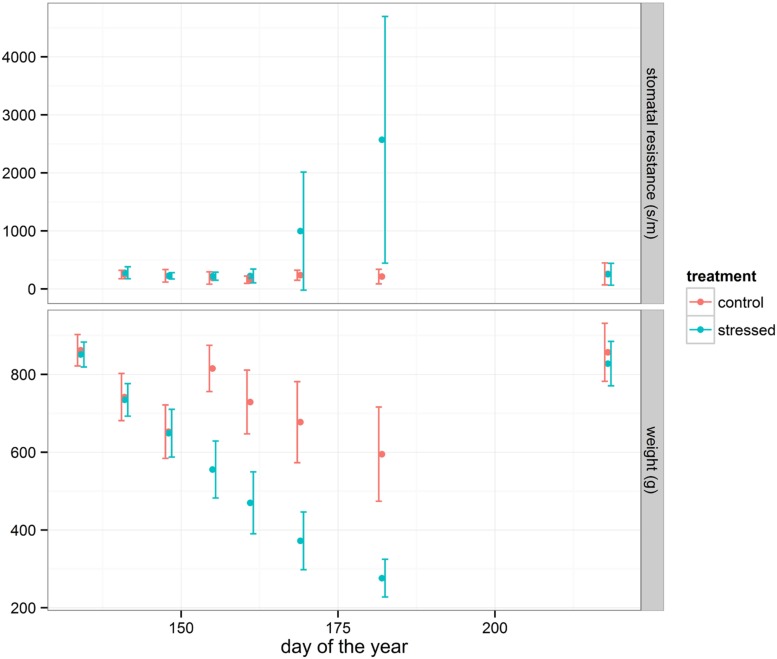
**Average stomatal resistance (subset of 30 plants for each treatment) and average weight loss (all pots) changes during the drought period**. Error bars (standard deviation) are shown.

In general, oak seedlings respond efficiently to drought stress by closing stomata, allowing the leaf water potential to remain above a critical threshold value at which cavitation damage occurs (Vivin et al., 1993; Cochard et al., 1996). To monitor the stress symptoms of the seedlings, stomatal resistance was measured during the drought period (**Figure 1**) in a sample of both the control and the drought-treated group of seedlings. The average value of the drought-treated group strongly increased from June 10, 2014 onward indicating a response to drought. At the same time, the average weight loss of the pots increased (**Figure 1**) confirming the drying process. Among the treated seedlings a small group of plants (23%) showed visual “wilting or curling of leaves” compared to the control group.

### Height Growth

**FIGURE 2:**
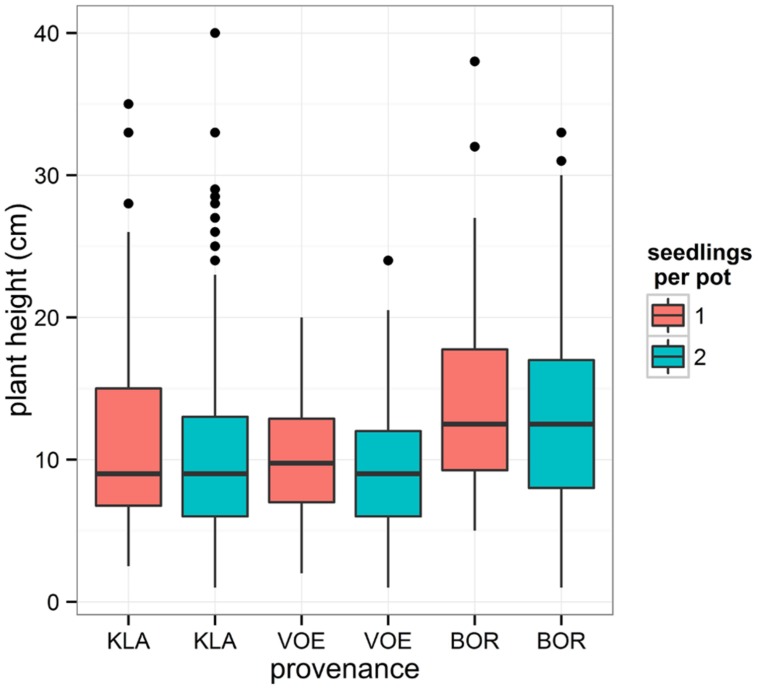
**Box plot showing the measured plant height at the end of the drought treatment for every provenance and according to the number of seedlings per pot**.

**FIGURE 3:**
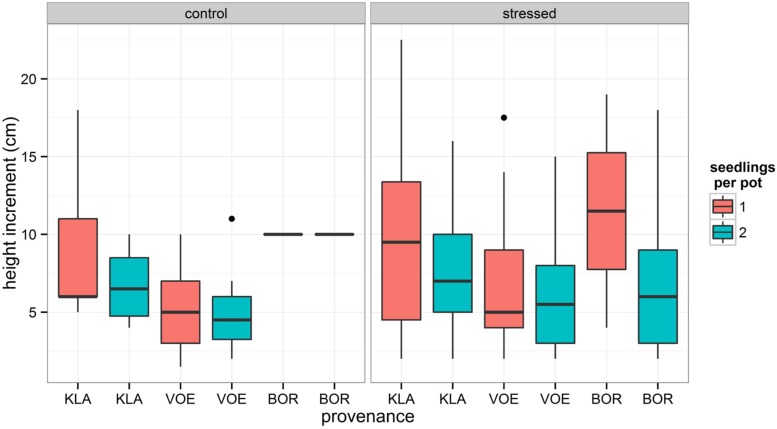
**Box plot showing the measured plant height increment during the recovery of the seedlings for both the control group and the drought treated seedlings, for every provenance and according to the number of seedlings per pot**.

**FIGURE 4:**
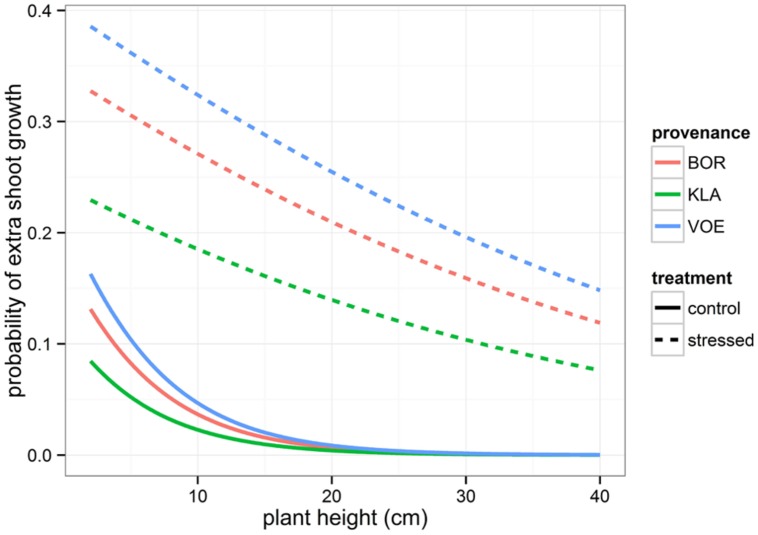
**Modeled probability for an extra shoot growth after the drought period, depending on the plant height at the end of the drought treatment and on the provenance**. Modeled probabilities are based on an average weight loss in the pots for the control and stressed group.

**FIGURE 5:**
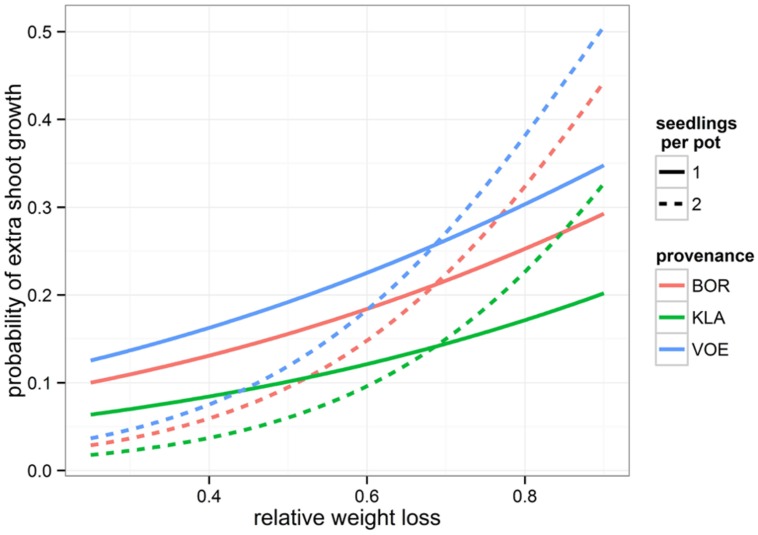
**Modeled probability of extra shoot growth after drought stress, depending on the relative amount of weight loss during the drought treatment (relative to the fully water saturated condition), the provenance of the oak seedlings and the number of seedlings per pot**. Modeled probabilities are based on an average height of 10 cm at the end of the drought period.

Boxplots of the first height measurement and of the height increment between the first and the second measurement are shown in **Figures 2** and **3**, respectively. The binary response variable indicating whether or not a seedling showed an extra height growth after re-watering, was modeled using generalized linear mixed models. Significant influencing factors were the provenance (no significant interaction term with weight loss), the number of seedlings per pot, depending on the amount of weight loss (significant interaction term) and the height of the seedlings at the end of the drought treatment, also depending on the amount of weight loss (**Table 2**). Both in stressed and control conditions the provenance VOE produced more extra shoot growth compared to KLA, with BOR in an intermediate position (**Table 2**, **Figure 4**). Especially seedlings that shared a pot displayed a higher probability for extra shoot growth in more stressed conditions (**Table 2**, **Figure 5**).

**Table 2:** Estimated coefficients for the fixed part of the logistic regression models with binary response variables extra shoot (Model I) and apical bud development (Model III).

Co-variable	Model I				Model III			
Co-variable	Extra shoot				Bud development			
	Estimated parameter	Std. error	*Z*-value	*P*-value	Estimated parameter	Std. error	*Z*-value	*P*-value
VOE	0.7454	0.3274	2.276	0.022^∗^	-0.6030	0.2851	-2.115	0.034^∗^
BOR	0.4917	0.4875	1.009	0.313	0.3338	0.4468	0.747	0.454
H	-0.2648	0.0826	-3.207	0.001^∗∗^	0.9100	0.4675	1.946	0.051
Wl	-1.0605	1.2530	-0.846	0.397	1.3228	1.0039	1.318	0.187
H:Wl	0.3081	0.1239	2.487	0.012 ^∗^	-2.5614	0.7644	-3.351	0.000^∗∗∗^
Sp	-2.0866	0.6708	-3.111	0.001^∗∗^				
Wl:Sp	3.0438	1.1500	2.647	0.008^∗∗^				

*The provenances VOE and BOR are compared to the standard provenance KLA. H: initial height of the plants before the start of the drought treatment; Wl: weight loss of plant pots relative to fully water saturated condition; Sp: number of seedlings per pot. Significance codes: ^∗∗∗^*P* ≤ 0.0001, ^∗∗^0.0001 < *P* ≤ 0.01, ^∗^0.01 < *P* ≤ 0.05.*

In addition, the higher the height at the end of the drought treatment, the lower the probability on extra shoot growth (**Table 2**, **Figure 4**).

**FIGURE 6:**
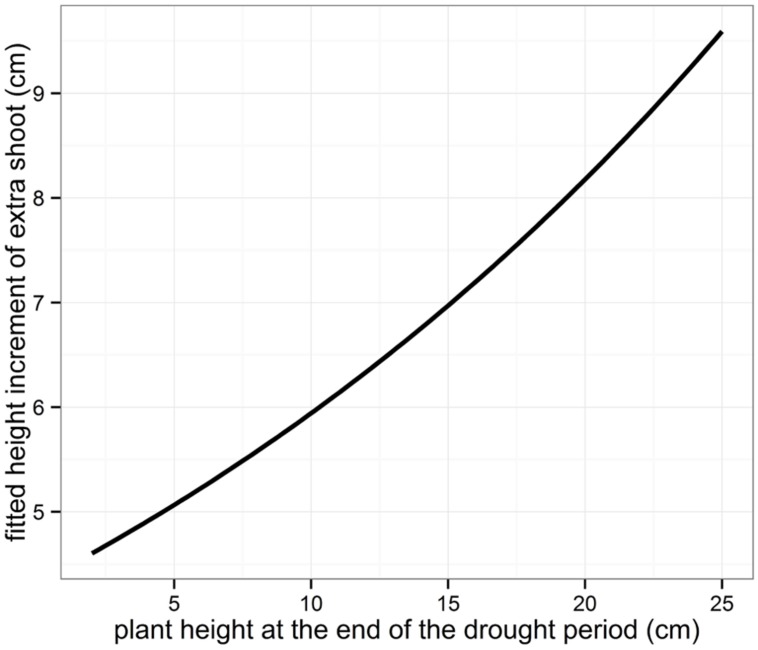
**Fitted height increment of the extra shoots grown after the drought period**.

For the seedlings showing extra shoot growth after re-watering, the actual length of the height increment was modeled using linear mixed models (**Table 3**, **Figure 6**.). The length of the extra growth was found to be only dependent on the plant height at the end of the drought treatment, with higher plants producing a larger extra shoot.

**Table 3:** Estimated coefficients for the fixed part of the linear model of height increment after the drought treatment.

	Estimated parameter	Std. error	*DF*	*T*-value	*P*-value
Intercept	1.463	0.1106	110	13.2231	0.000^∗∗∗^
H	0.0319	0.0098	110	3.2522	0.001^∗∗^

*H, height at the end of the drought treatment. Significance codes: ^∗∗∗^*P* ≤ 0.0001, ^∗∗^0.0001 < P ≤ 0.01.*

### Bud Development

**FIGURE 7:**
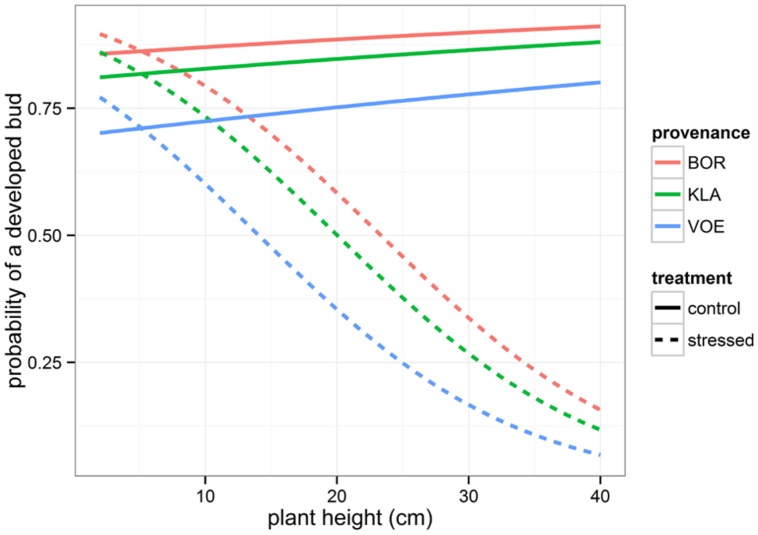
**Modeled probability of a fully developed apical bud 9 weeks after the drought treatment, depending on the plant height at the end of the treatment, on the treatment and on the provenance**. For the calculation of the modeled probabilities of control and stressed plants, the mean relative weight loss of the pots was applied.

Presence or absence of a fully developed apical bud in the fully recovered seedlings after the drought stress was monitored and the binary variable was modeled using generalized linear mixed models (**Table 2**, **Figure 7**). The drought-treated seedlings significantly showed less well-developed apical buds in higher plants (significant interaction term between initial height and relative weight loss). Both in drought-treated and control seedlings (no significant interaction term), VOE had a lower probability on a fully developed apical bud on the measurement day compared to the other provenances.

## Discussion

We studied the effect of a late spring/early summer drought stress on sessile oak (*Q. petraea*) seedlings originating from Belgium during the first growing year. In our experiment, potted oak seedlings responded with a higher probability on extra shoot formation after re-watering, especially for those seedlings that experienced direct competition for water (two plants per pot). Plant competition for water is less studied and not well understood compared to light and nutrient sources (Craine and Dybzinski, 2013). At the same time, the higher seedlings in the drought-treated group showed less well developed apical buds compared to the control group. Oak seedlings seem to translate the drought signal in an increased chance on extra shoot formation after re-watering. This reaction can be interpreted as a tendency to compensate fairly quickly for a retarded growth during the stress period, resulting in our experiment in the surprising and contra-intuitive higher average height increment in the drought-treated group of plants. Although only seedling height was measured, this result seems to contradict many descriptions of diminished biomass production of oaks after recovery from drought stress (Broadmeadow and Jackson, 2000; Thomas, 2000; Thomas and Gausling, 2000; Arend et al., 2011; Kuster, 2012), but it is in line with drought stress responses that led to the developments of extra shoots in oak (Spieß et al., 2012) and Douglas-fir (Kaya et al., 1994) after re-watering. Growth in Douglas-fir is characterized by a similar cyclic growth pattern as in oak (Kaya et al., 1994). Kaya et al. (1994) describe extra shoot production of Douglas-fir seedlings caused by re-watering after two drought periods, which took place 4 weeks long during the first growing season and 8 weeks long during the second growing season. Reich et al. (1980) describes conditions that favor multiple flushing of oak within one growing season. The observation that seedlings show more flushing per growing season compared to adult trees is related to the fact that an additional flush in adult trees would imply a relatively higher photosynthetic demand compared to seedlings. On the other hand, a higher root biomass in comparison to aboveground tissues, which has been studied in resprouting of coppiced stems, may allow a higher number of flushes within one growing season compared to seedlings. Also defoliation within the growing season has been reported to lead to extra flushing in oaks (Borchert, 1975). Even though the oak seedlings in our experiment only showed relatively minor leaf damage at the end of the drought treatment, and the majority of leaves fully recovered after re-watering, a stress signal indicating potential leaf loss may have triggered an extra flushing in a significantly higher number of seedlings compared to the control group, resulting in a larger mean height growth as side effect. Also, the extra shoot growth after re-watering can be considered as a compensation for the growth arrest experienced during the drought period (Spieß et al., 2012).

Our observed responses in primary height growth are contradictory to the observations of stress responses in secondary growth of adult oak trees as expressed in annual radial wood formation, which is dependent on cambial activity. Van der Werf et al. (2007) reported a retarded cambial activity during drought stress, which was not resumed after normalization of the conditions in the same growing season, which underpins the fundamental differences in regulation of primary and secondary growth in trees.

Two opposite response types of drought stress can therefore be suggested depending on the severity of the stress. Mild drought stress (no effect on stomatal conductance) resulted in less second flushes of *Q. petraea* (Thomas and Gausling, 2000) whereas our results indicate that short and more severe drought stress in early summer augmented the probability on a higher number of flushes, which is in line with the findings of Kaya et al. (1994).

In our experiment, the length of the extra shoot growth showed no difference between control and drought-treated plants, but was found to be larger for higher plants compared to smaller plants. Although the drought signal triggers a higher chance on extra shoot formation after re-watering, it does not influence shoot length as such. As the auxin/cytokinin ratio is one of the main regulators of bud dormancy and bud burst during the year (Cline and Harrington, 2006; Leyser, 2009; Müller and Leyser, 2011; Su et al., 2011; Vanstraelen and Benková, 2012), it can be hypothesized that drought stress signals act along this pathway.

In our experiment, drought-treated plants showed less fully developed and thus dormant apical buds among the higher seedlings 9 weeks after finishing the drought treatment compared to the control group, suggesting a height-dependent retardation in bud development. This effect seems to contrast with the higher chance on an extra shoot growth mainly among smaller plants, suggesting deviating signal pathways. Although independent of the level of drought stress, we observed significant differences between the studied provenances for extra shoot production and bud development. Compared to KLA seedlings, VOE and to a lesser extent BOR produced on average more shoots during our experiment independent from the treatment. More extra shoot growth later in the growing season may lead to a shortened hardening process in autumn, which increases the sensitivity to early frosts. Furthermore, compared to KLA, VOE seedlings showed less developed apical buds 9 weeks after re-watering, which may additionally increase the vulnerability of the seedlings during autumn and winter (Svejgaard Jensen and Douglas Deans, 2004). KLA seedlings represent a likely local provenance in the study region. Less shoot growth later in the growing season and quicker apical bud dormancy indicate a better adaptation to local climate, minimizing risks on frost damage both in well-watered and dry conditions.

## Conclusion

After an early summer drought event re-watering augmented significantly the probability to form an extra shoot in oak seedlings, with the highest probability in the smaller individuals. Simultaneously, drought retarded the apical bud development significantly in larger seedlings. Competition for water experienced by seedlings that grow together in a pot further increased the chance of extra shoot formation. As the number of extreme weather events will increase in the future due to climate change, it is important to study the behavior of seedlings subjected to more severe drought stress, because such experiments will greatly assist us in understanding the impact of drought on forest regeneration.

## Author Contributions

AT conducted the experiment with KM and wrote the introduction and parts of the discussion and conclusion. KM was the main supervisor during the project AT, KM, MM, and GM conducted the statistical analyses, created the graphs, and integrated them in the article. MM worked on graph 2. GM worked on graph 3. KS established the experimental design, monitored the observations, reviewed the manuscript, and wrote parts of the discussion. ES and ÉE worked on the seedling evaluation systems, filtered the data, and evaluated it. ES and ÉE wrote the material and methods parts. All authors participated in the finalization of the article.

## Conflict of Interest Statement

The authors declare that the research was conducted in the absence of any commercial or financial relationships that could be construed as a potential conflict of interest.
